# Clinical application of genetic testing for posterior uveal melanoma

**DOI:** 10.1186/s40942-016-0030-2

**Published:** 2016-02-01

**Authors:** Victoria J. Schopper, Zelia M. Correa

**Affiliations:** University of Cincinnati College of Medicine, Medical Arts Building, 222 Piedmont Avenue, Suite 1500, Cincinnati, OH 45209 USA

**Keywords:** Uveal melanoma, Choroidal melanoma, Primary ocular tumors, Prognostic testing, Gene expression profiling, GEP, Multiplex ligation-dependent probe amplification, MLPA

## Abstract

Uveal melanoma is the most common primary intraocular tumor in adults, and it has a strong potential to metastasize. Traditionally, clinicopathological features of these tumors were used to provide a limited prediction of the metastatic risk. However, early genetic studies using karyotype analysis, fluorescence in situ hybridization, and comparative genetic hybridization of posterior uveal melanoma samples identified multiple chromosomal abnormalities associated with a higher risk of fatal metastasis. This correlation between specific genetic abnormalities in uveal melanoma and a patient’s risk for development of metastasis has recently been widely studied, and the development of new prognostic tests has allowed clinicians to predict this metastatic risk with increased accuracy. Such novel tests include gene expression profiling, which analyzes the RNA expression patterns of tumor cells, and multiplex ligation-dependent probe amplification, which detects deletions or and amplifications of DNA in tumor cells. This review discusses the current status of prognostic testing techniques available to clinicians and patients for posterior uveal melanomas.

## Background

**Fig. 1:**
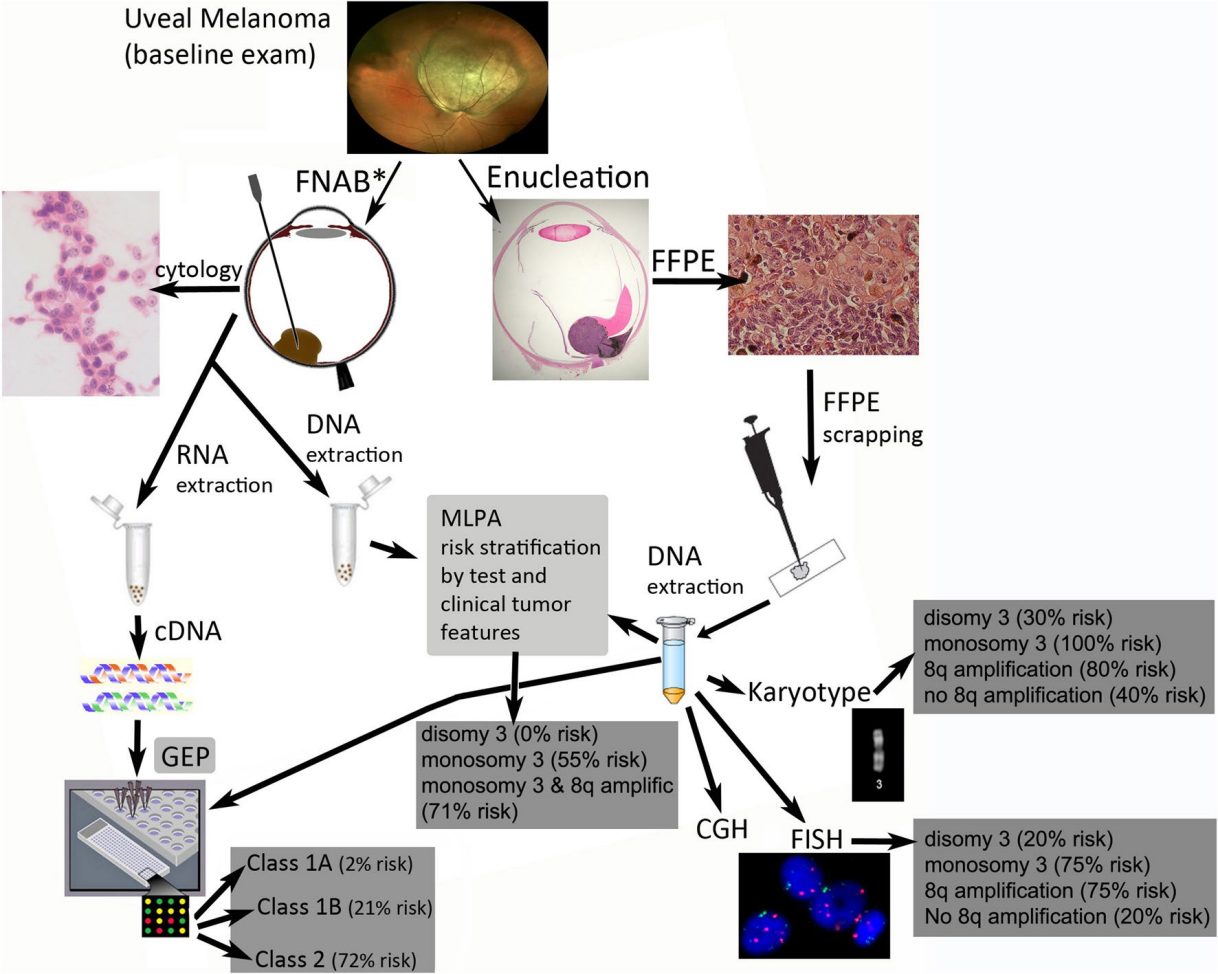
Posterior uveal melanoma prognostic test flow-chart. Because the current prognostic tests rely on either DNA or RNA extraction from tumor specimens. Tumor tissue procurement should be done ideally prior to any form of local destructive treatment that may alter the DNA and/or RNA of the tumor cells (including radiation). Tissue can be obtained from either an enucleation specimen that has been formalin-fixed and paraffin-embedded (FFPE) or a fine needle aspiration biopsy (FNAB) of the tumor prior to conservative treatment or immediately after enucleation. FFPE scrapings from an enucleation specimen can be sent for DNA extraction and can allow for further analysis of the tumor through karyotyping, FISH (fluorescence in situ hybridization), CGH (comparative enomic hybridization), MLPA (multiplex ligation-dependent probe amplification), or GEP (gene expression profiling). Through FNAB, small quantity of fresh tumor cells is extracted from the tumor. These cells can be sent for cytology, GEP, or MLPA. GEP relies primarily on RNA extraction from these cells but it can also be performed using DNA. MLPA relies solely upon DNA extraction. GEP stratifies tumors into Class 1A, Class 1B, or Class 2 based 12 discriminating genes and 3 control genes. MLPA yields a complex report describing risk stratification of the test that includes the genetic information yielded, clinical features of the tumor, and patient demographics. Estimated 10-year metastasis-free survival is listed based on publications on karyotype analysis [9], FISH [12], MLPA [13]. Estimated 5-year metastasis-free survival based on GEP classification is also listed [7]

Posterior uveal melanoma (PUM) is the most common primary tumor of the eye, and it carries a high risk for metastasis, primarily to the liver. Approximately 50 % of patients with PUM will develop fatal metastases, and many patients will die within a year of their metastatic diagnosis [1]. Often, by the time metastatic disease is detectable on imaging studies, the tumor burden is substantial and therapeutic options are limited [2]. Given the potentially aggressive nature of these tumors, 97 % of patients reportedly desire prognostic information in order to make decisions about management and surveillance testing for metastasis from their uveal melanoma [3]. Traditionally, demographic and clinicopathologic features of these tumors have been used to determine the metastatic risk of a PUM. Such features as older patient age, larger tumor basal diameter, invasion of the sclera, ciliary body involvement, and epithelioid cell type have been associated with worse patient prognosis and a higher incidence of metastatic disease [4]. Although these identified risk factors are still widely used, their accuracy to predict metastatic potential has been shown to be limited. With the rapid development of new molecular techniques that allow for examination of the genetic make-up of tumor cells, more accurate prognostic information has become available to clinicians and patients. This prognostic information allows for personalized clinical decision-making and hopefully more targeted therapeutic options. Novel techniques such as gene expression profiling (GEP) and multiplex ligation-dependent probe amplification (MLPA) have improved upon the initial methods for genetic testing like karyotype analysis, fluorescence in situ hybridization (FISH), and comparative genomic hybridization (CGH). Although some of the methods described herein are not easily accessible throughout the world, it is important that physicians are up to date with the latest advances in the prognostication of PUM. In this manuscript, the authors review the recent developments and current status of prognostic testing of patients with PUM, what these tests analyze, and how their results can be applied in clinical practice (Fig. 1).

### Karyotype analysis

In the early 1990s, initial genetic studies of PUM examined the implications of a gain or loss of specific chromosomes in these tumors. Through karyotype analysis, Prescher et al. discovered that monosomy 3 and increased copies of chromosome 8q were commonly found in PUM samples [5]. Further studies by these authors demonstrated an increased potential for metastatic disease in patients with such chromosomal abnormalities. According to the authors, patients who retained both copies of chromosome 3 exhibited no metastatic disease within the median follow-up time of 3.4 years, whereas 57 % of patients with monosomy 3 developed metastases [6]. Given its prognostic importance, monosomy 3 has subsequently remained the focus of many genetic studies [7]. In addition to monosomy 3, a gain in chromosome 8q has also been linked with poor survival prognosis [8]. Karyotype analysis has been proven accurate for tumors with obvious gain or loss of entire chromosomes, but it failed to detect minor genetic changes. Further, the 10-year reported mortality of patients with disomy 3 was 30 % and those with monosomy 3 loss was 100 %; patients with no chromosome 8q amplification was 40 %, and those with chromosome 8q amplification was 80 % [9]. However, karyotype analysis can additionally provide false negative results in cases of isodisomy 3 found in some PUM. A study by White et al. identified uveal melanomas that had developed acquired homozygosity of chromosome 3, otherwise known as isodisomy 3. In short, during their progression, such tumors lose one copy of chromosome 3, and start duplicating their remaining third chromosome. On karyotype analysis, the duplicated genetic material remains undetected even though the tumors demonstrate functional monosomy [10].

### Comparative genomic hybridization

Comparative genomic hybridization (CGH) is a technique that helps to identify a gain or loss of chromosomal material within tumor DNA. Normal DNA and tumor DNA are labeled with different fluorescent probes and subsequently hybridized. An increase or decrease in the color ratio of the fluorescent probes identifies areas of abnormal chromosomal material. While it has been used to study the cytogenetics of PUM, CGH fails to detect partial deletions or smaller defects in the tumor DNA [11].

### Fluorescence in situ hybridization

Fluorescence in situ hybridization (FISH) is method for detecting the gain or loss of chromosomal material that can be used in several different tissue samples including PUM. FISH uses labeled fluorescent probes that match specific DNA target sequences. Through FISH, changes in the DNA copy number of these sequences can be determined in both fresh tissue samples and paraffin-embedded tumor specimens. Analysis of tumor samples using FISH has identified monsomy 3 and amplification of 8q in PUM to be associated with poor disease-free 10-year survival in a study by van den Bosh et al. [12]. Although FISH has been shown to be accurate in detecting gain or loss of larger DNA sequences similar to CGH, it often misses minor chromosomal defects or aberrations. Thus, it has limited utility in making a precise clinical prediction in patients who have no detectable loss or gain of chromosomal material [13].

### Gene expression profiling

As a relatively novel technique, gene expression profiling (GEP) can be used for rapid detection of the up-regulation or down-regulation of particular genes of interest in minute tissue samples. The technique involves the isolation of RNA from a tissue sample, followed by conversion to cDNA that is subsequently hybridized to genechips, and microarray analysis is performed. Through GEP assay of untreated PUM tissue samples, Harbour and associates demonstrated two distinct prognostic classes that can be used to predict metastatic risk and strongly correlates with patient survival [14]. The authors initially identified 62 genes that showed distinct aberrant expression patterns. When combined with the clinical outcome of patients, the up-regulation or down-regulation of specific gene clusters identified by the GEP assay allowed for this new stratification scheme. Patients with class 1 tumor gene expression profiles have low-grade tumors with a decreased risk of metastatic spread. On the other hand, patients with Class 2 tumor gene expression profiles have high-grade tumors with an increased tendency to metastasize [14]. When compared to the presence of monosomy 3 and the clinical and pathologic tumor features, GEP demonstrated superior accuracy at predicting the risk of metastatic disease in patients with PUM [4]. While both CGH and FISH provide a snapshot in time of the genetic make-up of the tumor cells, because GEP is an RNA based assay, it predicts how the tumor cells are likely to behave as far as metastatic spread.

Following the initial study of GEP in PUM samples, Harbour and associates developed a 15-gene PCR based assay that enabled discrimination of Class 1 and Class 2 tumors. The assay examines the expression patterns of 12 class discriminating genes identified by the previous analysis and 3 control genes shown to be unchanged in uveal melanomas [2]. Although GEP can be performed in paraffin fixed tissue, tumor cell procurement for this test is mainly done by fine needle aspiration biopsy (FNAB) at the time of plaque implantation or immediately after enucleation of the eye containing the tumor. The fresh tumor aspirate obtained is flushed in a buffer solution and then frozen until the time of the test. The assay was then tested in a multicenter prospective clinical trial in the Collaborative Ocular Oncology Group Report Number 1, where it correctly classified tumors in 97.2 % of cases [15]. Now commercially available as DecisionDx-UM^®^ (Castle Biosciences, Tucson, AZ), the assay can be used routinely to provide patients with prognostic information about their tumors [16]. Since the development of the assay, Class 1 tumors have been further subdivided in Class 1A and Class 1B. Class 1A tumors have a 2 % 5-year metastatic risk while Class 1B tumors have a 21 % 5-year metastatic risk. By contrast, Class 2 tumors have a 72 % 5-year metastatic risk. This prognostic information has had significant implications for the ongoing clinical monitoring of patients with uveal melanomas [7]. A study by Correa et al. determined that the GEP assay provides genomic information even in very minute specimens using fine needle aspiration biopsy aspirates. According to the study, only 0.6 % of all 159 cases had a failed result [17].

Interestingly, in very small tumors, heterogeneous genetic make-up could potentially limit the accuracy of this test (and likely any other test used in samples obtained by needle aspirates) in a small number of patients. In a study by Augsburger et al. tumor sampling from different sites within the same tumor demonstrated discordance of GEP classification in 11.3 % of cases [18]. However, the authors noted a correlation between the thickness of the tumor and GEP discordance between biopsies. The study showed discordant GEP results were found in 23.8 % of tumors that were less than 3.5 mm thick, 16.7 % of tumors between 3.5 and 7 mm thick and only 4.3 % of tumors thicker than 7 mm. The authors concluded that performing a GEP on a biopsy sample from a single tumor site carries a risk of prognostic misclassification that is significant in smaller tumors. This study recommend taking this information into account when advising patients with smaller tumors about their prognosis [18]. Meanwhile, the authors are working on a prospective study looking at GEP discordance in small PUM.

### Multiplex ligation-dependent probe amplification

Schouten et al. described a method termed multiplex ligation-dependent probe amplification (MLPA) that can be used for detecting the relative quantities of as many as 40 different DNA sequences [19]. MLPA analyzes the gain and loss of chromosomal material. Through the reaction, denatured genomic DNA is mixed with probes for the specific target genes of interest. The probes each consist of two oligonucleotides that will hybridize to adjacent sites on the target DNA sequences. One of these oligonucleotides contains a stuffer sequence of unique length. Following hybridization, the probes are ligated and amplified by PCR. The individual stuffer sequences give each probe a unique length that enables effective separation via electrophoresis. The separated PCR products can be quantified and the amount extrapolated to determine the relative expression of the gene products. MLPA is sensitive and sequence specific in detecting changes in DNA copy numbers and detects deletions and amplifications of single exons [19]. MLPA can be performed on fresh frozen and formalin-fixed and paraffin-embedded tissue samples, although fresh frozen samples are preferable [20].

The application of MLPA to PUM tissue samples was subsequently described by Damato et al. Their initial study analyzed uveal melanoma tissue samples from 73 patients. MLPA detected chromosomal abnormalities that correlated with metastatic death, most importantly loss of chromosome 3 and gain of chromosomal material on 8q [21]. In a larger study from the same group, chromosomes were considered abnormal if any of the loci examined showed either borderline or definite gain or loss [13]. The results showed that chromosome 1p loss, chromosome 3 loss, and chromosome 8q gain correlated with increased mortality whereas chromosome 6p gain correlated with improved survival. The mortality rate was 0 % in 133 patients without any evident chromosome 3 loss and 71 % in patients with both monosomy 3 and 8q gain with a median follow up time of 1.89 years. The MLPA results additionally correlated with the clinicopathological features of the tumors. Through this study, MLPA provided relevant prognostic information related to chromosomal aberrations to patients with uveal melanomas. While the MLPA results of this study provided accurate prognoses, it does not offer similar stratification of cases as GEP and prognostication requires detailed interpretation by the clinician ordering the test and it can still be improved by clinicopathologic correlation [13]. MLPA is now commercially available through Impact Genetics, Toronto, Canada.

Based upon previous evidence of genetic heterogeneity, Dopierala et al. examined a larger number of cases to determine the percentage of intratumor heterogeneity seen in formalin-fixed paraffin-embedded PUM through MLPA. Their results indicated that 75 % of the tumors showed heterogeneity in one or more loci on chromosomes 1, 3, 6, or 8. The authors of the study conclude that an MLPA result from a single random formalin-fixed and paraffin-embedded sample from a PUM may not provide an accurate representation of the tumor’s genetics. In contrast to studies using GEP, MLPA identified a higher percentage of tumors with a heterogeneous genetic make-up in this study [22].

### Mutational profiling

Although not useful in prognostication of patients, detection of specific mutations in uveal melanomas may lead to improved therapeutic options in the future. Through a search for mutations in the oncogenic pathway involving RAF, MEK, and ERK, Onken et al. first identified a mutation in the stimulatory alpha(q) G protein subunit known as GNAQ in approximately 50 % of PUM samples. A mutation in GNAQ was detected in PUM samples at all of the stages of malignant progression, indicating that such a mutation may play a role in the initial development of the tumor [23]. Van Raamsdonk et al. discovered that mutations in the protein GNA11, a paralogue of GNAQ, were found in 32 % of PUM samples and 57 % of PUM metastases. Furthermore, the authors found that mutations in GNA11 were sufficient to induce metastases in a mouse model. Mutations in GNAQ and GNA11 affect a critical oncogenic signaling cascade that affects the metastatic potential of tumors [24]. Ewens et al. demonstrated that identification of specific mutations may have prognostic significance when combined with the chromosome 3 status of the tumor. In their study of 63 cases of PUM, GNA11 and BAP1 mutations were associated with a greater metastatic risk while a mutation in EIF1AX was associated with a lower metastatic risk within the 48 months of follow up [25]. These mutations may provide targets for therapeutics. Specific inhibitors, such as the MEK inhibitor selumetinib, can be designed to interrupt the pathways of these oncogenic proteins and may have important clinical implications [7].

## Discussion

While relatively new, prognostic testing is becoming an important component in the metastatic risk stratification of patients with PUM. Understanding the information obtained by each test and its application is fast becoming an important part of ocular oncology clinical practice. Table 1 briefly highlights features of different prognostic tests currently used for uveal melanoma. Although sensitive in detecting large chromosomal abnormalities such as monosomy 3, karyotype analysis, FISH, and CGH have largely been replaced by more recently developed prognostic tests like GEP and MLPA. GEP analyzes the RNA expression patterns of the tumor cells and provides a precise classification for metastatic risk. Whilst it carries a modest risk of mis-classification due to possible genetic heterogeneity in smaller tumors, it is the most robust independent predictor of metastatic risk for patients with uveal melanoma. MLPA detects deletions and amplifications of DNA in tumor cells, and it offers information about the common chromosomal abnormalities associated with metastatic risk in PUM. As MLPA identifies high rates of genetic heterogeneity in PUM, particularly in formalin-fixed and paraffin-embedded samples, correlation with the clinical and pathologic features of the tumors seems essential in providing patients with accurate information and makes test interpretation more complex and prognostication less clear. Further, the reported failure rate of MLPA is higher than GEP.

**Table 1:** Comparison of laboratory tests currently available for prognosis of posterior uveal melanomas

	Karyotype	FISH	CGH	GEP	MLPA
Type of analyses	Chromosomes	DNA	DNA	RNA or DNA	DNA
Monosomy 3 detection	Yes	Yes	Yes	No	Yes
Tissue used	FFPE	FFPE	FFPE	FFPE or fresh	FFPE or fresh
Reported tumor heterogeneity	NR	NR	NR	11.3 %	75 %

*FISH* fluorescence in situ hybridization; *CGH* comparative genomic hybridization; *MLPA* multiplex ligation-dependent probe amplification; *GEP* gene expression profiling; *FFPE* formalin fixed paraffin embedded; NR not reported

In short, there is significant discussion about which one of these is the best prognostic test for uveal melanoma? In the authors’ opinion, the best prognostic test is one with the highest negative predictive value and fewer prognostic subgroups. Based on this premise, GEP should be the preferred prognostic test because it has the aforementioned features and is the only one validated prospectively in a multi-center clinical trial [15]. Whenever GEP testing is not available; FISH and MLPA provide comparable chromosomal information [26]. Because the accuracy of FISH and MLPA is not as high as GEP it is recommended that additional clinical information including patient demographic information, tumor features, histo and/or cytopathology be considered to improve their prognostic precision [27]. Centers where neither of these tests is available can resort to cytology for prognosis [28] but should also add clinical information to improve their prognostic accuracy [29].

## Conclusions

Although the therapeutic options currently available for metastatic PUM are limited, patients still desire accurate prognostic information about their tumors at the time of diagnosis in order to plan their management. In the future, more standardized metastatic surveillance for those patients found to have aggressive tumors might allow for earlier detection of metastatic disease and improved clinical management. While these prognostic tests have not yet led to new therapies for PUM, they provide a critical step in the direction towards identifying specific therapeutic modalities that target the genetic abnormalities of these tumors.

## Abbreviations

PUM: posterior uveal melanoma
GEP: gene expression profiling
MLPA: multiplex ligation-dependent probe amplification
CGH: comparative genomic hybridization
FISH: fluorescence in situ hybridization
